# Minimally Invasive Esthetic Reinforcement of a Fractured Anterior Tooth Using Dentapreg PINPost: A Case Report

**DOI:** 10.7759/cureus.60303

**Published:** 2024-05-14

**Authors:** Aanchal Banka, Gaurav Patri, Debkant Jena, Harshita Lath, Geonath Immanuel

**Affiliations:** 1 Department of Conservative Dentistry and Endodontics, Kalinga Institute of Dental Sciences, Bhubaneswar, IND

**Keywords:** fiber-reinforced composite post, reinforcement, maxillary central incisor, endodontically treated tooth, dentapreg pinpost

## Abstract

The reason to use post is to retain the core that holds the definitive prosthesis. The maxillary central incisor always poses a challenge during reconstruction using the post and core system. Dentapreg PINPost, a pre-cured flexible post made of fiber-reinforced composite (FRC), has many advantages over metallic posts and fiber posts. The purpose of this case report is to present an innovative technique to place both FRC posts and FRC sheets as a single assembly into the canal which gives a monoblock effect. This technique is feasible and may eradicate some of the problems associated with the failure of the post and core.

## Introduction

The fracture resistance of teeth that have undergone root canal therapy is influenced by iatrogenic variables, which include dehydration of dentin, chemicals, medications, and hard tissue loss.

Reeh et al. reported that endodontic treatments have a minimal impact on tooth stiffness, reducing it by only 5%, although restorative and access cavity operations lead to a 20% reduction [1]. Recently, Plotino et al. underscored the significance of structural deterioration in access cavities, demonstrating that traditional access cavities notably decrease fracture resistance compared to more conservative approaches [2]. Hence, it is crucial to reinforce teeth intracoronally to safeguard them against fractures [3,4], particularly in posterior teeth with compromised mesial and distal walls, where occlusal forces can lead to cusp fractures if left unprotected. Therefore, root canal-treated teeth exhibit structural disparities from untreated vital teeth and necessitate specialized restorative interventions.

The advancement of fiber-reinforced composite (FRC) technology has led to increased utilization of fiber materials in extensive dental restorations. Typically composed of polyethylene and glass fibers, FRC exhibits a comparable elastic modulus to dentin and sufficient strength for effective oral function. The arrangement of polyethylene woven fiber within coronal restorations can influence the fracture resistance of the restoration [5]. Oskoee et al. demonstrated a significant increase in fracture resistance by altering the fiber position, shifting it from the gingival surface to the occlusal surface [6].

Clinical research indicates a notably high success rate ranging from 95% to 99% for teeth restored using FRC posts [7]. These have gained recognition for their ability to enhance aesthetics, replicate dentin-like physical properties, and offer superior biomechanical performance. Nevertheless, in instances where the canal is extensive and irregular, these posts may not seamlessly conform to the tapered shape of the canal. Consequently, excessive cement may be needed to bridge the gap between the post and the remaining dentin, potentially leading to irregularities between the two [8]. A recent approach involving multiple-pin posts has been introduced to tackle these challenges to enhance post-adaptation within the canal.

Dentapreg® (Dentapreg; ADM, Brno, Czech Republic) has invented pioneering pin posts for tooth reconstruction after endodontic procedures. These posts are characterized by their innovation, minimal invasiveness, rapidity, and safety. They are designed to be flexible, enabling them to conform precisely to the natural contour of the root without necessitating additional instrumentation. This flexible design concept preserves the tooth’s original biomechanics, thereby reducing the risk of root fracture [9].

In this case study, a fractured anterior tooth was successfully rehabilitated using a combination of pin posts and zirconia crowns in the anterior esthetic zone.

## Case presentation

A 27-year-old male patient presented to the Department of Conservative Dentistry and Endodontics with a chief complaint of a broken tooth in the upper front tooth region as a result of falling on the floor the night before. The patient did not have any relevant past dental or medical history. On clinical examination, there was a soft tissue laceration of the lip which was sutured in the Department of Oral and Maxillofacial Surgery, following which he was referred to the Department of Conservative Dentistry and Endodontics. Upon radiographic examination, widening of the periodontal ligament space was observed without disruption of the lamina dura. Vitality testing utilizing an electric pulp tester revealed a significantly low pain threshold for the tooth. Therefore, the diagnosis rendered was symptomatic apical periodontitis, concomitant with an Ellis Class III fracture concerning tooth 11. The treatment plan, encompassing root canal therapy followed by the placement of a post and core and subsequent crown restoration, was thoroughly explained to the patient, who consented to proceed with the proposed intervention.

Clinical steps

In the first visit, access opening and BMP were done till F3 (Dentsply Sirona, York, PA, USA). Calcium hydroxide intracanal medicament was given for 10 days. On the second visit, after sectional obturation, 3-5 mm of gutta-percha at the apex of the root was applied and the canal was rinsed with water and dried carefully with paper points. The depth of the prepared canal was measured using the endodontic instrument. The canal was treated with a self-cure bonding agent (Solare universal bond, GC, Japan) and was light-cured. Subsequently, the canal was filled with dual-cure resin cement (Allcem core, FGM, USA) from the apex part and continued slowly upward until the whole canal was filled. Now, Dentapreg PINPost from the storage bottle was taken out and coated with a thin layer of dual-cure resin cement.

**Figure 1:**
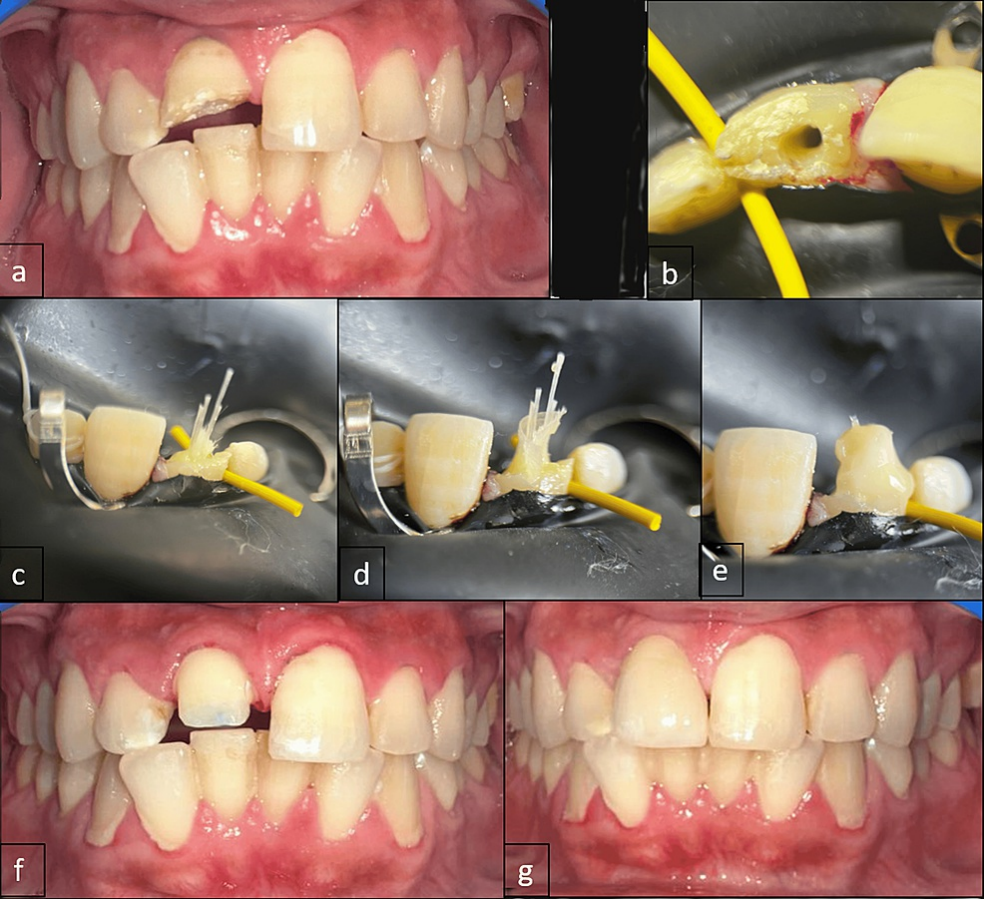
(a) Clinical picture of tooth 11 with Ellis Class III fracture. (b) Clinical picture of tooth 11 showing access opening. (c) Clinical picture of tooth 11 with multiple Dentapreg PINPosts. (d) Clinical picture of tooth 11 showing Dentapreg UFM sheet wrapped around the pinposts. (e) Clinical picture of the post-assembly and composite build-up before finishing. (f) Clinical picture of crown preparation. (g) Clinical picture of tooth 11 post-crown cementation.

**Figure 2:**
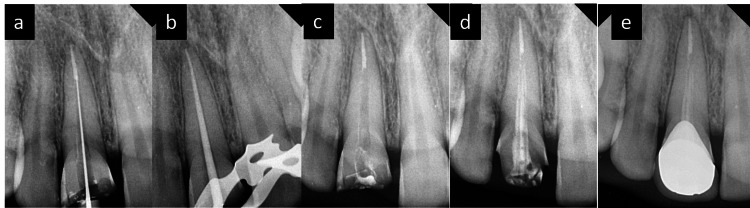
(a) Intraoral periapical (IOPA) radiograph showing the working length determination of tooth 11. (b) IOPA radiograph showing the master cone fit in tooth 11. (c) IOPA radiograph showing sectional obturation in tooth 11. (d) IOPA radiograph of tooth 11 showing the placement of Dentapreg PINPosts assembly inside the canal and cemented using dual-cure resin cement. (e) IOPA radiograph of tooth 11 post-crown cementation.

The Dentapreg PINPost was slowly inserted into the canal, and this step was repeated depending on the canal’s width. Dentapreg® UFM strip from the blister was trimmed using scissors to the necessary length and wrapped around the exposed Dentapreg PINPost on the coronal portion. Flowable composite (G-Anieal, GC, Japan) was used to manipulate the Dentapreg® UFM strip and then light-cured for 40 seconds. The core was built using the same dual-cure resin cement. After finishing the crown preparation, intraoral scanning was done to take the impression, and the shade and the crown were fabricated. On the third visit, the zirconia crown was luted using resin-modified glass ionomer cement (3M RelyX Luting Plus Resin Modified Glass Ionomer Cement, USA). All steps are explained in Figures 1a-1g and Figures 2a-2e.

## Discussion

Post-endodontic restorations have always been a clinician’s dilemma. The primary cause of failure in the metal post and core systems typically involves post loosening, followed by root fracture, caries, abscess formation, bent or fractured posts, and root perforations.

Traditionally, prefabricated posts have been available, which can be either cemented or bonded. However, both methods require careful preparation of the root canal space to ensure sufficient retention and resistance to dislodgment. There has been an ongoing debate among researchers regarding the impact of removing root dentin for these prefabricated tapered posts, as it can lead to the propagation of micro-cracks or excessive weakening of the inner radicular dentin, potentially leading to future complications.

FRC posts have made it easier to restore teeth that have undergone endodontic treatment but still have structural compromise. Because FRC posts are made of glass and polyethylene and possess an elastic modulus similar to that of dentin. They strengthen structurally damaged roots and improve bonding. Furthermore, the ability of FRC posts to transmit light helps the luting materials to cure throughout the whole length of the tooth, leading to a noticeably stronger push-out bond [10].

In this study, Dentapreg multiple fiber pin posts were utilized. These posts form a monoblock with the tooth. They are stiff but adaptable, consisting of slender S-type unidirectional glass fiber pins with a 0.45 mm diameter that were initially created to meet the space shuttle’s strength requirements. Dentapreg PINPosts adapt to the contour of the root canal without the need for additional instrumentation. They offer multi-scale retention, including micromechanical retention due to the increased number of pin posts and the use of adhesive agents [9].

Multiple smaller pin posts, when compared to a single broader post of similar length, exhibit a higher modulus of elasticity. Placing several posts across a wider surface area helps restrict crack propagation by distributing tensile stress over a larger area. Studies by Li et al. and Fráter et al. indicate that using numerous FRC posts is advantageous for compromised roots compared to using single fiber posts [11,12].

The effectiveness of fiber post-retention is likely impacted by various adhesives. The GC SOLARE Universal Bond, utilized in this research, stands out as a comprehensive and distinct bonding agent suitable for all etching methods. Its exceptional bond strength to tooth structure, encompassing both enamel and dentin, is attributed to its unique formulation. The dimethacrylate monomer component in SOLARE Universal Bond contributes to its porosity in both enamel and dentin, distinguishing it from other bonding agents. Conversely, the elevated concentration of phosphate ester monomer enhances the etching process [13,14].

Self-etch adhesives streamline the bonding process by combining conditioning and priming steps. These adhesives have acidic monomers that demineralize and infiltrate the dental substrate simultaneously, eliminating the requirement for a separate etching step. Consequently, this approach is touted as more user-friendly and less technique-sensitive [11].

Another important consideration is the elevated “configuration factor” produced in post spaces during polymerization of luting cement. When this fast shrinkage is combined with little flow relief, many interfacial gaps may occur. Therefore, the choice of cement for luting posts is crucial. Dual catalyst cement with chemical activation has been suggested to counteract this shrinkage resulting from rapid polymerization [11]. In our study, dual-cure resin cement (Allcem core, FGM, USA) was selected as the luting cement due to its reputation for delivering uncompromising results, high bond strength, and long-lasting esthetics according to the manufacturer’s claims.

It is worth noting that while in vitro studies offer valuable insights, they have restrictions and are unable to entirely substitute clinical trials. Further clinical investigations are warranted to achieve more promising outcomes.

## Conclusions

This case report presented a novel post and core material to conserve root dentin and provide more fracture resistance. Long-term follow-ups, however, are still necessary and should be the main focus of future research. In addition, more in vitro and ex vivo research is needed to assess if the fracture pattern produces favorable or unfavorable fractures.
